# Some Remarks upon Cases of Obstruction of the Bowels, in Which Surgical Means Were Used

**Published:** 1871-03

**Authors:** A. Reeves Jackson

**Affiliations:** 785 Michigan Avenue; Chicago, Ill.


A MONTHLY RECORD OF

*Medicine, Surgery, and the Collateral Sciences.*

EDITED BY J. ADAMS ALLEN, M.D., LL.D.; AND WALTER HAY, M.D.

---

VOL. XXVIII.—MARCH, 1871.—NO. 3.

---

### Original Communications.

ARTICLE I.—*Some Remarks upon Cases of Obstruction of the Bowels, in which Surgical means were used.* By A. REEVES JACKSON, M.D., Chicago, Ill.

CASE I. *Strangulation of ileum by a band of adhesion—Puncture of Intestine—Gastrotomy—Recovery.*

The patient was a woman aged twenty-eight years. She had been married four years, but had never borne children. Menstruation commenced at thirteen, and had always been painfully performed. During the preceding fifteen months she had suffered from occasional attacks of apparently causeless constipation, accompanied by colicky pains, and followed by diarrhœa. Sometimes during these painful seizures she had experienced a degree of relief by getting upon her elbows and knees. At first these attacks were separated by an interval of six to eight weeks, but latterly, that is to say, within the past four or five months, they had become more frequent, the interval between some of them not exceeding two weeks. Besides, during the past ten months she had been suffering from a cough, which had become more and more troublesome, and in that period had had two slight attacks of hæmoptysis. Still she had kept up, and was able to do ordinary household work.

The present illness had commenced on the morning of Oct. 2d, 1859, with sharp pain in the left iliac region, which was attributed to the ingestion of some improper article of diet. The pain was more constant than it had been on any previous occasion, and was increased by pressure. Without advice, she had taken a full dose of some purgative pills, and finding them ineffectual, at the end of five hours the dose was repeated. As these also failed to produce the desired effect, an ounce of castor oil was taken, and immediately vomited. From this time forward almost everything was returned that was taken into the stomach. The pain increased in severity, and was no longer confined to the spot in which it originated, but extended upward, and into the right side. The abdomen became swollen and its walls tense. A physician being called at this stage of the case, prescribed a turpentine enema, which brought away a small quantity of dark brown fæces, without relief to the symptoms. A second enema of warm water was then administered, but it came away in a few minutes unchanged. There being no facilities for giving a warm bath, the patient was then wrapped in blankets wrung out of warm water, and a teaspoonful of laudanum administered. This was retained nearly half an hour, and was then vomited, together with a draught of water, which she had imprudently taken. The dose of laudanum was repeated, and this time retained. The patient now became easier, and was drowsy. A purgative enema, consisting of an infusion of senna with sulphate of magnesia, was then given, and firm pressure made against the anus to prevent its return. Its pressure produced so much pain, however, that it was permitted to escape. All active treatment was now suspended. The patient was kept under the influence of opium, while small quantities of beef tea and brandy were given from time to time.

I saw her Oct. 4th, the third day of her illness. At this time she was in a state of semi-consciousness, and almost unable to speak. Her extremities were cold; pulse small, feeble, and rapid. Nausea and ineffectual retching were constant. The stomach had not retained anything during the past twelve hours. Her face, bathed in cold sweat, was pale and haggard, and its expression denoted that life was fast ebbing. The abdomen was enormously distended, and the writhing of the enlarged coils of intestine could be distinctly seen through the walls which enclosed them. The rectum was empty and contracted. I was informed that the urine had been scanty, high-colored, and voided without difficulty.

With scarcely a hope of doing more than giving temporary relief, I introduced a small exploring trocar and canula through the walls of the abdomen, at a prominent point, into the distended bowel. This gave immediate exit to a large quantity of flatus, and the abdominal fulness rapidly subsided. Flannels, saturated with a hot infusion of hops, were applied so as to envelope the entire abdomen; sinapisms to the calves of the legs and epigastrium; and a teaspoonful of brandy, with a fourth of a grain of morph. sulph. was administered. The patient had become somewhat aroused, and expressed a sense of relief. A stomach tube was now passed into the colon and a large quantity of beef tea very gradually injected, but, despite every effort to prevent its return, it was expelled almost immediately. At the same time the patient vomited the brandy, and this was followed by eructations having a decidedly stercoraceous odor, although there was no fœcal matter thrown up. The tympanites returned also, and in an hour the abdomen appeared as large and resonant as it had been prior to the puncture.

I now proposed opening the abdomen, in the folorn hope that something might yet be done to overcome the obstruction which evidently existed, and the operation was decided upon.

Commencing a little below the umbilicus, an incision was made in the linea alba. and continued directly downwards three inches. On opening the abdominal cavity the omentum was brought into view, its lower edge being forced through the incision by the pressure of the distended bowel. The cut was now lengthened about an inch, when a large coil of intestine, very greatly congested, protruded through the wound. Taking hold of this, I commenced the work of tracing up the point of obstruction. This was soon accomplished. Making gentle traction upon the bowel, I found that it could not be drawn from behind the omentum, and upon raising the latter upward and passing a finger beneath it, the cause was apparent. It consisted of a band of lymph, about two inches in length and nearly half an inch in width, extending from the posterior free surface of the omentum to the lower side of the transverse colon, forming a triangular space between these parts. A fold or loop of the ileum had insinuated itself through the opening thus formed, and becoming enlarged and congested, was unable to return. The band, which was tolerably firm in texture, was ruptured by the fingers, and the strangulated bowel freed. The portion which had thus been strictured was of a dark purplish color, while marks of incipient inflammation of the peritoneal investment of the small intestine above this point were everywhere present. I had the satisfaction of observing a very great change take place in the appearance of the liberated bowel before closing the wound. It became immediately filled with flatus, now able to pass onward, and its color changed from purple to dark red. I searched for and found the spot which had been punctured by the trocar. It was about fifteen inches above the point of strangulation. It formed the centre of a deep red space nearly an inch in diameter. The opening was no longer perceptible, and I was unable to discover the escape through it of either gas or fluid. I removed the whole of the adventitious band which caused the obstruction, and then, after carefully replacing the parts, closed the wound with four points of twisted suture. A flannel compress dipped in warm water was applied over the wound, and confined by means of a flannel binder, loosely pinned. During the time this was being done, large quantities of gas escaped per anum, and they were soon followed by a copious discharge of fluid fœces. Strict rest was enjoined, and a full dose of opium given.

The wound healed well, but the operation was followed by an attack of peritonitis, which was treated by opium, quinine, and a concentrated nourishing diet, and from which she slowly recovered. Nearly five weeks elapsed before she could be considered fairly convalescent.

This patient died of phthisis pulmonalis, in September, 1860, eleven months after the operation.

CASE 2. *Cancerous Disease of Sigmoid flexure—Artificial Anus—Death four months after.*

Thomas L., a farmer, aged forty-seven years, while ploughing, felt a pain in the left iliac region. At first it seemed slight, but soon became so severe as to oblige him to cease work. On reaching home, he passed a solid motion, with partial relief. The pain soon returned as violently as before, and he took a dose of laxative medicine, which aggravated the pain, but produced no other result. His general health had been good, and his bowels regular until within the past two months, during which time there had been a tendency to constipation. During this period, also, he had experienced an occasional vague sense of uneasiness in the lower part of the abdomen, and sometimes this had amounted to actual pain, but it had never before become so severe, and had always been relieved by an action of the bowels. Towards evening he vomited. For eight days he grew gradually worse. In this time he had taken several doses of purgative medicine, and had a number of laxative enemata. The bowels had not acted at all. The urine had been abundant and was quite natural in appearance. The pain had extended over the whole abdomen, which had become very much distended. Vomiting had been frequent, but among the watery and bilious substances ejected, there had been nothing noticeably stercoraceous.

He came under my notice May 11, 1861—the ninth day of his illness. At this time he was very restless, and continually changing his position. He seemed to be suffering a great deal of pain, the pain and tenderness extending generally over the abdomen, which was greatly distended, especially at the umbilicus. Pulse 108, small and compressible; respiration quickened; countenance anxious; tongue thickly covered with a dark yellow coating.

A flexible tube was passed as far as possible into the bowel—about twelve inches—and a quantity of warm water thrown slowly in. It was retained about half an hour and then came away unchanged. Flannels wrung out of hot water, sprinkled with turpentine, were applied to the abdomen, and forty drops of laudanum administered, the latter to be repeated every three hours. Finding that the stomach would not retain this, suppositories, containing each two grains of opium, were substituted.

12th. 10 A. M. Symptoms much the same. There has been no action of the bowels. Pain rather less—a fact due probably to the effect of the opium. Pulse 120; distension of the abdomen about the same. Has had eructations with a fœcal odor, but no vomiting since last evening.

In the afternoon his condition became more critical. Vomiting had returned, and was now unmistakeably stercoraceous. The pain was diminished, but his countenance assumed a peculiar leaden hue, and he expressed much anxiety as to his state, which he had not previously done. I proposed the formation of an artificial anus, and, having fully explained to the patient the nature and effects of the operation, readily obtained his consent.

Accordingly, having brought him fully under the effect of an anæsthetic mixture, consisting of one part chloroform and two of sulphuric ether, by measure, he was placed in a suitable position and the usual operation of Amussat performed on the right side.

An incision four inches in length was made parallel with the crest of the ilium, midway between it and the lower rib, commencing at a point opposite the sacro-lumbalis muscle. This was extended downwards until the three abdominal muscles, together with the anterior portion of the latissimus dorsi and a part of the quadratus lumborum were divided. A quantity of fatty tissue found at the bottom and back part of the wound, was torn away with the fingers and the handle of the scalpel, and the colon brought into view. It was readily distinguished, both by its fixedness and its characteristic greenish color. It was very greatly distended, and bulged slightly into the wound. A curved needle, furnished with a stout double thread, was passed through it, including about one inch between the points of entrance and emergence. Some of the cellular attachments of the bowel were then carefully separated, and by the aid of the thread it was drawn into the centre of the incision. A roll of cotton wool was packed around the bottom of the wound for the purpose of preventing the occurrence of fœcal extravasation. A transverse incision half an inch long was then made in the colon, and gave exit to a large quantity of fluid fœces and flatus. The distension of the abdomen rapidly subsided, and its walls became soft and supple. There could now be felt in the left iliac fossa a distinct hardness, occupying a space half as large as the open hand. The incision in the bowel was now increased to about two inches, and complete clearance having been effected, the edges were carefully attached to those of the integument at the posterior part of the wound, by means of silver wire sutures. The remainder of the wound was closed by hare-lip pins, with a view to obtaining union by first intention. Water dressing was applied, and strict rest enjoined.

The patient recovered well from the operation, but never fully regained his health, and died four months after.

With much difficulty, permission was obtained to make a post mortem inspection. In the upper part of the hollow of the sacrum, to the left side, was found an irregular hard mass, involving the lower two-thirds of the sigmoid flexure. It had completely obliterated the calibre of the bowel, so that I was unable to pass a small probe through without breaking the tissue. Ulceration had commenced at the upper part of the growth, and a small quantity of offensive, grayish pus was present. Above the seat of disease the bowel was thickened for a distance of nearly five inches, extending into the transverse colon. A similar condition was found also in the bowel below the growth, involving a portion of the rectum. A number of tubercles, varying in size from a pin's head to a pea, were observed in the neighborhood of the growth. On cutting into the diseased mass, the section presented a striated appearance, was of a whitish-yellow color, and varied much in consistence in different portions, being hard and dense in some places and soft in others—the latter condition obtaining most largely in the immediate vicinity of the ulcerated spot. Scattered throughout all parts of the growth, were to be seen isolated masses of transparent, gelatinous colloid. The cancerous character of the disease was beyond doubt. By request of the friends, the other organs of the body were not examined.

785 Michigan Avenue.

(To be continued.)

